# The Repair of Large Gaps in Peripheral Nerves by Neuroplasty

**Published:** 1919-01

**Authors:** 


***The Repair of Large Gaps in Peripheral Nerves by Neuroplasty.*** By KENNETH A. J. MACKENZIE. *Surgery, Gynecology and Obstetrics*, October, 1918.

The author reports three cases illustrating the utilization of nerve flaps of both central and peripheral origin in order to bridge unusually large gaps in peripheral nerves.

In one case quoted, regeneration occurred over a gap 10 3/4 inches in length; the sciatic was involved in a large malignant tumor, and the whole trunk of the nerve had to be excised over a length 10 3/4 inches. In two successive operations, performed at an interval of 46 days, flaps 16 inches long, were cut from both the internal and external popliteal nerves, and their ends were implanted into clefts previously cut in the stump of the sciatic nerve. Both flaps were tucked in separate muscular beds. In both cases the wounds healed by first intention.

The following results were obtained :

(1) Trophic recovery of the leg practically complete.

(2) Almost universal recovery of deep sensation.

(3) Recovery of motion and power in groups of muscles which, after the excision of the nerve, were reduced to a complete paralytic state.

(4) Direct sensibility of the new track of the nerve to deep pressure, and the transmission of painful sensibility thereby to the foot.

(5) Independent and unaided locomotion.

The second case illustrates the utilization of central instead of peripheral flaps.

As a result of a gunshot wound becoming infected, so much scar tissue developed as to lead to the destruction of 4 inches of the sciatic trunk and 3 inches of both the two branches. Two flaps containing 6 or 8 nerve fibers, 8 inches in length, were detached from both divisions of the sciatic trunk and anastomosed to the popliteal branches.

Considering the fact that infection was virulent and prolonged, and the case a most desperate one when the operation was attempted, and also that extensive regenerative changes had taken place in the leg, it may be assumed that, by timely operation and the use of sound central flaps, regeneration and recovery of function would undoubtedly have been complete.

The third case illustrates the possibility of bringing about recovery of function in a divided nerve in the presence of infection in which there is discharging sinus and bone necrosis.

The patient suffered from an ununited, infected fracture of the right humerus. In addition to the fracture, there was complete division and paralysis of the musculospiral nerve. After excising the scar tissue, the gap in the nerve proved too long to allow of the approximation of both ends. Accordingly a flap, 1 1/2 inches long, was made from the central end. This was used to bridge the gap, and was sutured to the distal end of the nerve which had previously been cross-sectioned until it showed normal nerve fibres. End results were excellent; fifteen months later the patient was able to take up the massage course in war service.

The author adds that unimpaired nerve tissue should always be utilized for the effective repair of damaged nerves, and that, in their repair, nerves can be successfully sequestrated in muscular tissue so as to promote their own regeneration and that of the muscles in which they are embedded.

